# Endothelial cell biomechanical adaptation to altered connexin 43 expression under fluid shear stress

**DOI:** 10.1242/jcs.264023

**Published:** 2026-08-14

**Authors:** Md Mydul Islam, Vignesharavind Subramanianbalachandar, Robert Steward

**Affiliations:** ^1^ Department of Mechanical and Aerospace Engineering, University of Central Florida, USA; ^2^ Burnett School of Biomedical Sciences, University of Central Florida, Orlando, FL 32816, USA

**Keywords:** Connexin 43, Tractions, Intercellular stress, Endothelial cell, Fluid shear stress

## Abstract

We examined how pharmacological modulation of connexin 43 (Cx43; also known as GJA1) regulates endothelial biomechanics under static conditions and laminar fluid shear stress (FSS). Cx43 activity was either inhibited with 2′,5′-dihydroxychalcone (chalcone) or enhanced with retinoic acid (RA), and biomechanical responses were quantified by measuring tractions, intercellular stress, and cell velocity. Low-dose chalcone increased tractions and intercellular stresses under both static and FSS conditions, whereas high-dose chalcone attenuated these stresses. In contrast, low-dose RA reduced tractions and intercellular stresses under static conditions but enhanced them under FSS, while high-dose RA consistently suppressed mechanical stresses. Chalcone treatment caused dose-dependent reductions in F-actin stress fibers and a reduction in gap junction intercellular communication (GJIC). RA treatment preserved actin organization and increased GJIC up to a threshold concentration, beyond which GJIC declined. Together, these findings demonstrate that Cx43 may regulate endothelial biomechanics in a dose-dependent and flow-dependent manner, supporting a role for gap junctions in endothelial mechanoadaptation to fluid shear stress.

## Teaser

**Summary:** Pharmacological modulation of connexin 43 modulates endothelial tractions and intercellular stresses in a dose- and flow-dependent manner, highlighting a role for gap junctions in endothelial mechanoadaptation to fluid shear stress.

## INTRODUCTION

Endothelial cells (ECs) generate contractile forces to constantly probe their microenvironment (Gordon et al., 2020), adhere to their underlying extracellular matrix (van Hinsbergh and Koolwijk, 2008), sense cell shape and cell size (Hosseini et al., 2015), and propel themselves through their environment (Al-Soudi et al., 2017). In addition, dynamic collective migration gives rise to intercellular stresses within a cell monolayer owing to the physical connection between neighboring ECs (Fournier et al., 2010; Sieminski et al., 2004). While EC tractions are generated by actin–myosin interactions that are transmitted to the extracellular matrix via focal adhesion complexes and integrins, intercellular stresses are propagated through the monolayer via mechanosensitive cell–cell junctions (De Pascalis and Etienne-Manneville, 2017; Gulino-Debrac, 2013; Schwarz and Soiné, 2015; Trepat et al., 2009). Endothelial tight junctions and adherens junctions have been proposed to be primarily responsible for endothelial barrier permeability and barrier strength because of their reported roles as mechanosensors (Bazzoni and Dejana, 2004; le Duc et al., 2010; Liu et al., 2010; Nielsen et al., 2012; Wallez and Huber, 2008). However, gap junctions have recently been suggested to play a role in barrier permeability (Fournier et al., 2010; Schwarz and Soiné, 2015) and barrier strength (Gulino-Debrac, 2013), but the role of endothelial gap junctions in the endothelial biomechanical response is poorly understood.

Gap junctions physically connect the cytoplasm of adjacent cells and provide a path for ions and specific biomolecules (<1 kDa) to travel between neighboring cells (Figueroa and Duling, 2009; Nielsen et al., 2012; Söhl and Willecke, 2004). ECs primarily express connexin 40 (Cx40; also known as GJA5), connexin 37 (Cx37; also known as GJA4) and connexin 43 (Cx43; also known as GJA1) (Haefliger et al., 2004; Márquez-Rosado et al., 2012; Söhl and Willecke, 2004). Alterations or deletions in any of the gap junctions listed above have been reported to result in severe vascular complications. Cx43 is one of the most important endothelial gap junctions due to its abundance within the vasculature (Haefliger et al., 2004; Kwak et al., 2002; Márquez-Rosado et al., 2012). In addition, several studies have linked abnormal Cx43 expression to EC dysfunction, a precursor to cardiovascular disease. For example, Cx43 expression has been reported to increase and assist in the early stage of atherosclerosis in mice (Kwak et al., 2002), overexpression of Cx43 has been shown to increase vascular permeability in rats (Zhang et al., 2015), age-related downregulation of Cx43 in atrial tissues of guinea pigs has been suspected to promote the progression of atrial fibrillation (Nagibin et al., 2016), and Cx43 deficiency has been reported to cause dysregulation of vasculogenesis in mice (Walker et al., 2005). In addition, the C-terminus of Cx43 protein has been reported to interact with the cytoskeleton and other cell junctional proteins, indicating a possible influence of Cx43 on EC mechanics (Okamoto et al., 2019). Taken together, the reports mentioned above suggest that alterations in Cx43 expression may significantly impact endothelial biomechanical function and potentially contribute to the progression of vascular disease.

Our group previously demonstrated that reduced Cx43 activity by 2′,5′-dihydroxychalcone (chalcone) influences endothelial biomechanics in a dose-dependent manner by causing an initial increase and subsequent decrease in endothelial tractions and intercellular stresses (Islam and Steward, 2019a,b). Intercellular stresses are mechanical stresses generated between cells in a monolayer, and tractions are the stresses generated between cells and their underlying substrate via actomyosin contractility. To investigate how connexin 43 (Cx43) influences endothelial mechanobiology, we treated human umbilical vein ECs (HUVECs) with chalcone or retinoic acid (RA) to pharmacologically inhibit or enhance Cx43 activity, respectively, and examined their responses under static conditions and laminar fluid shear stress (FSS). We subsequently measured tractions and intercellular stresses using traction force microscopy (Butler et al., 2002; Tambe et al., 2011) and monolayer stress microscopy (Tambe et al., 2013), respectively. Our traction force microscopy and monolayer stress microscopy results revealed that under both static and FSS conditions, ECs exposed to a low dose of chalcone yielded higher tractions and intercellular stresses compared to cells exposed to a high dose of chalcone under static conditions. In addition, low-dose RA reduced endothelial tractions and intercellular stresses relative to untreated and high-dose RA-treated monolayers under static conditions, while low-dose RA-treated ECs under FSS yielded higher tractions and intercellular stresses than high-dose RA-treated monolayers and untreated monolayers. We also tested the impact chalcone and RA had on gap junction intercellular communication (GJIC), filamentous actin (F-actin) structure and Cx43 structure of human aortic ECs (HAECs). We observed actin stress fibers and Cx43 structure to become less visible, and GJIC to gradually diminish with increasing chalcone concentration, while F-actin and Cx43 structure appeared to remain relatively unchanged and GJIC activity increased with increasing RA concentration up until a threshold beyond which GJIC activity decreased. We believe our results highlight the importance of Cx43 in endothelial biomechanical response.

## RESULTS

### Dose-dependent effects of chalcone on endothelial biomechanics under static and FSS conditions

**Fig. 1.**
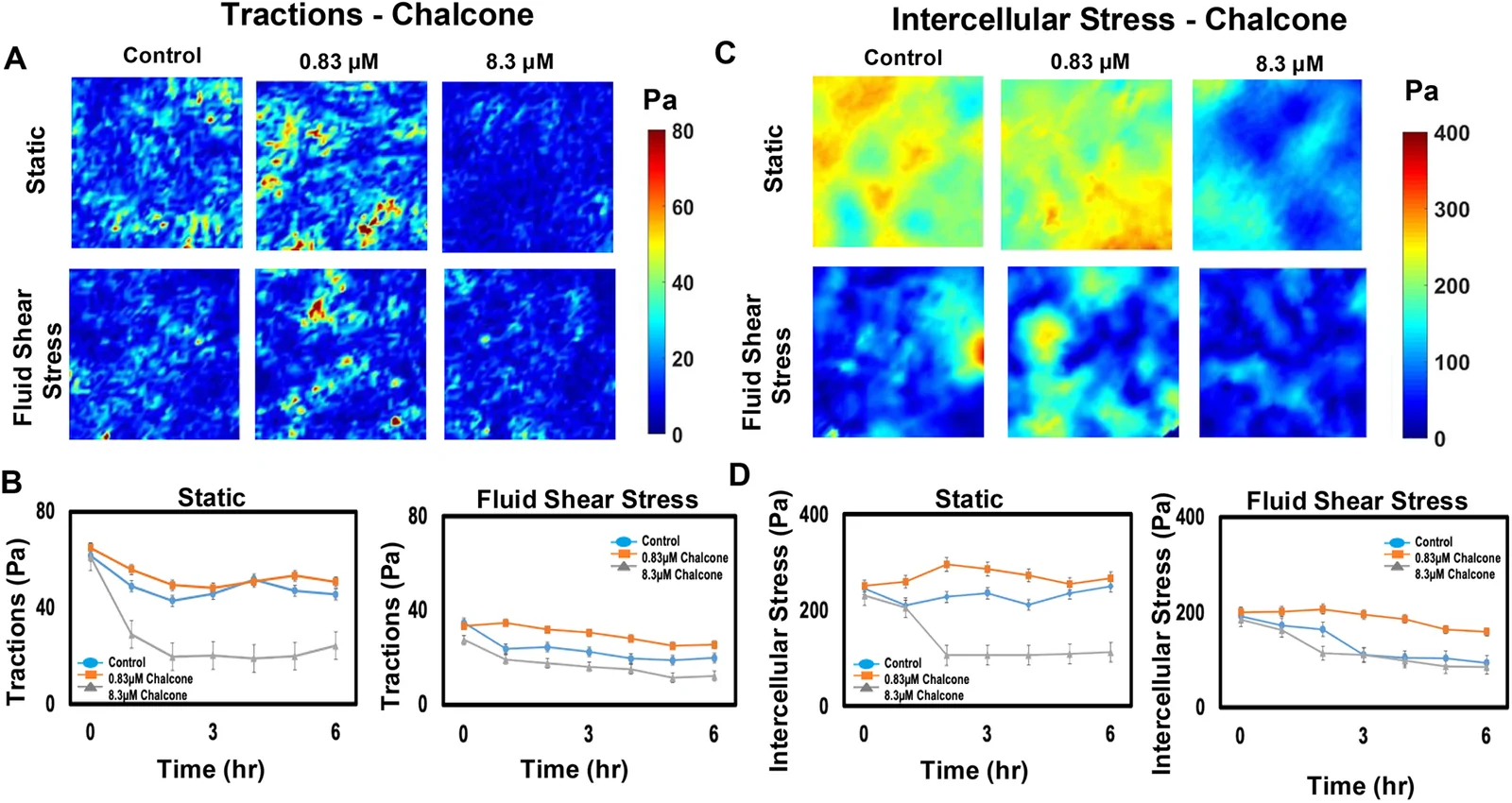
**Impact of chalcone on tractions and intercellular stress.** (A,B) Traction color plots of HUVECs exposed to vehicle control and chalcone concentrations of 0.83 μM and 8.3 μM under static and FSS conditions (A) and tractions versus time plot of HUVECs exposed to chalcone under static and FSS (B). (C,D) Intercellular stress color plots of HUVECs exposed to vehicle control and chalcone concentrations of 0.83 μM and 8.3 μM under static and FSS conditions (C) and intercellular stress versus time plot of HUVECs exposed to chalcone under static and FSS (D). Traction color plots (A) and intercellular stress color plots (B) are shown at the end of a 6 h imaging period, which was preceded by a 1 h chalcone pretreatment. Error bars are s.e.m. (*n*=30 monolayers). Statistical significance was determined by performing a single-factor ANOVA analysis. (Tractions: PANOVA=9.08×10^−10^<0.05 for static and PANOVA=4.2×10^−12^<0.05 for FSS conditions; intercellular stress: PANOVA=2.27×10^−07^ for static conditions and PANOVA=0.000722<0.05 for FSS conditions).

To assess the impact of Cx43 inhibition on endothelial biomechanics, we measured tractions and intercellular stresses generated by HUVEC monolayers treated with chalcone under static and FSS conditions. Under static conditions, low-dose chalcone (0.83 μM) treatment resulted in a 22% increase in tractions (56±5 Pa; mean±s.e.m.) compared to untreated controls (46±4 Pa) (Fig. 1A,B). In contrast, high-dose chalcone (8.3 μM) treatment led to a 56% decrease in tractions (20±7 Pa) (Fig. 1A,B). Similarly, under FSS, low-dose chalcone treatment increased tractions by 28% (26±2 Pa) compared to controls (20±1 Pa), while high-dose chalcone treatment decreased tractions by 39% (12±1 Pa) (Fig. 1A,B). Traction intercellular stresses followed a similar trend, with low-dose chalcone increasing intercellular stresses by 7% (265±20 Pa) under static conditions (Fig. 1C,D) and by 69% (158±5 Pa) under FSS (Fig. 1C,D) compared to controls (249±14 Pa and 94±4 Pa, respectively). High-dose chalcone reduced intercellular stress by 55% (112±17 Pa) under static conditions and by 9% (85±4 Pa) under FSS.

Strain energy measurements further supported these findings, with low-dose chalcone increasing the strain energy by 87% and 82% under static and FSS conditions, respectively, whereas high-dose chalcone decreased the strain energy by 77% and 32%, respectively (Fig. S1A,B).

### RA modulates endothelial biomechanics in a flow-dependent manner

**Fig. 2.**
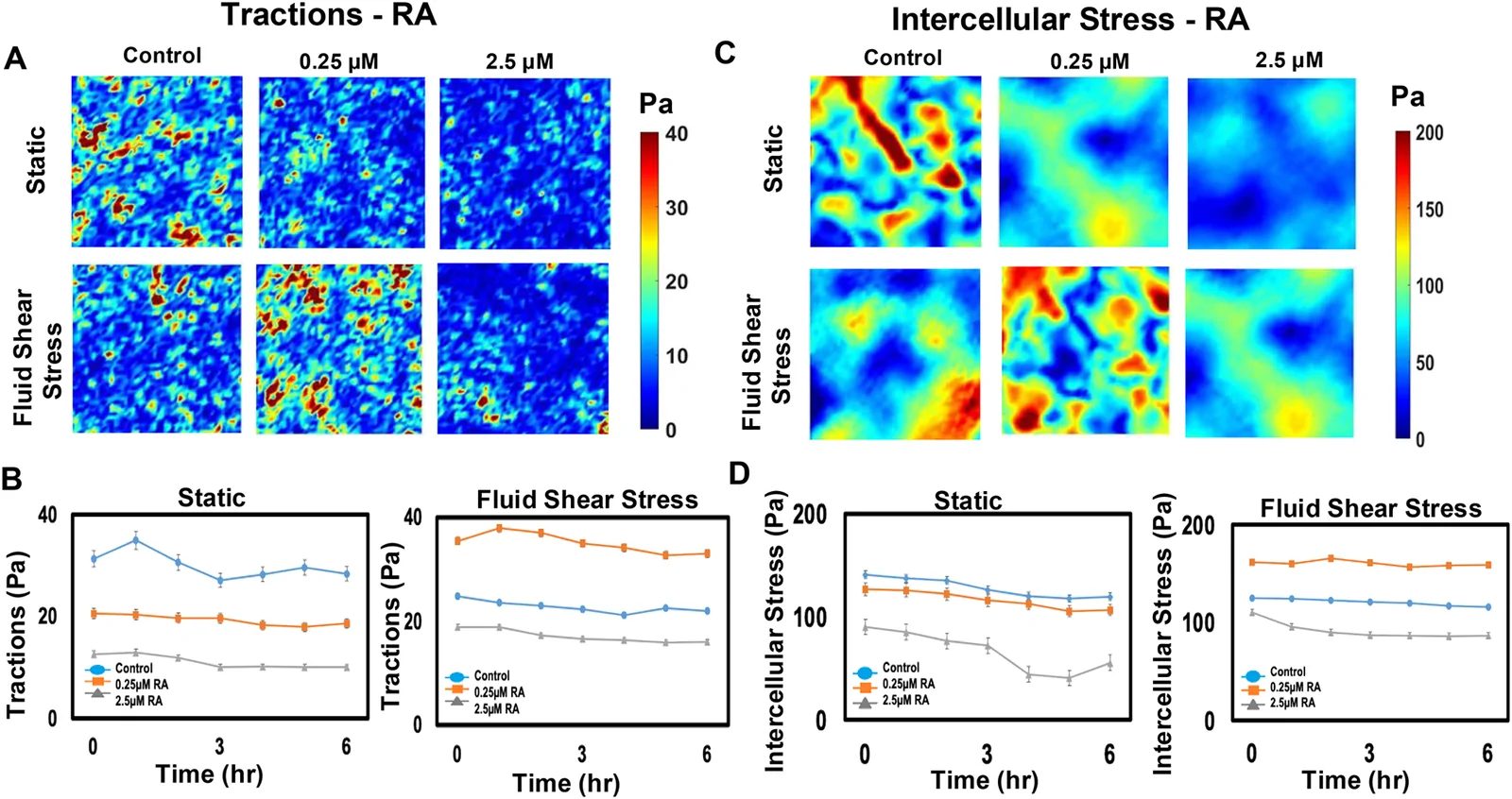
**Impact of RA on tractions and intercellular stress.** (A,B) Traction color plots of HUVECs exposed to vehicle control and RA concentrations of 0.25 μM and 2.5 μM under static and FSS conditions (A) and tractions versus time plot of HUVECs exposed to chalcone under static and FSS (B). (C,D) Intercellular stress color plots of HUVECs exposed to vehicle control and RA concentrations of 0.25 μM and 2.5 μM under static and FSS conditions (C) and intercellular stress versus time plot of HUVECs exposed to RA under static and FSS (D). Traction color plots (A) and intercellular stress color plots (B) are shown at the end of a 6 h imaging period, which was preceded by a 24 h RA pretreatment. Error bars are s.e.m. (*n*=30 monolayers). Statistical significance was determined by performing a single-factor ANOVA analysis. (Tractions: PANOVA=8.3E^−11^ for static and PANOVA=4.09×10^−12^<0.05 for FSS conditions; intercellular stress: PANOVA=5.24×10^−07^<0.05 for static and PANOVA=1.64×10^−5^<0.05 for FSS conditions).

To investigate the effects of Cx43 upregulation on endothelial biomechanics, we treated HUVEC monolayers with RA and quantified cellular tractions and intercellular stress under static and FSS conditions. Under static conditions, both low-dose (0.25 μM) and high-dose (2.5 μM) RA decreased tractions by 34% (19±6 Pa) and 65% (10±3 Pa), respectively, compared to controls (28±5 Pa) at 6 h following a 24 h drug pretreatment (Fig. 2A,B). In contrast, under FSS, low-dose RA increased tractions by 51% (33±4 Pa) compared to controls (22±3 Pa), while high-dose RA decreased tractions by 27% (16±3 Pa) (Fig. 2A,B).

Intercellular stresses exhibited a pattern similar to tractions. Low-dose RA caused a decrease of 11% (107±8 Pa) under static conditions, but induced an increase of 37% (159±4 Pa) under FSS compared to controls (120±7 Pa and 116±4 Pa, respectively) (Fig. 2C,D). High-dose RA consistently reduced average normal intercellular stresses by 53% (56±4 Pa) under static conditions and by 26% (87±4 Pa) under FSS (Fig. 2C,D).

Strain energy calculations revealed a 29% decrease and 64% increase in strain energy with low-dose RA treatment under static and FSS conditions, respectively, while high-dose RA decreased the strain energy by 55% and 29%, respectively (Fig. S1C,D).

### Cx43 perturbation affects endothelial morphology and migration

**Fig. 3.**
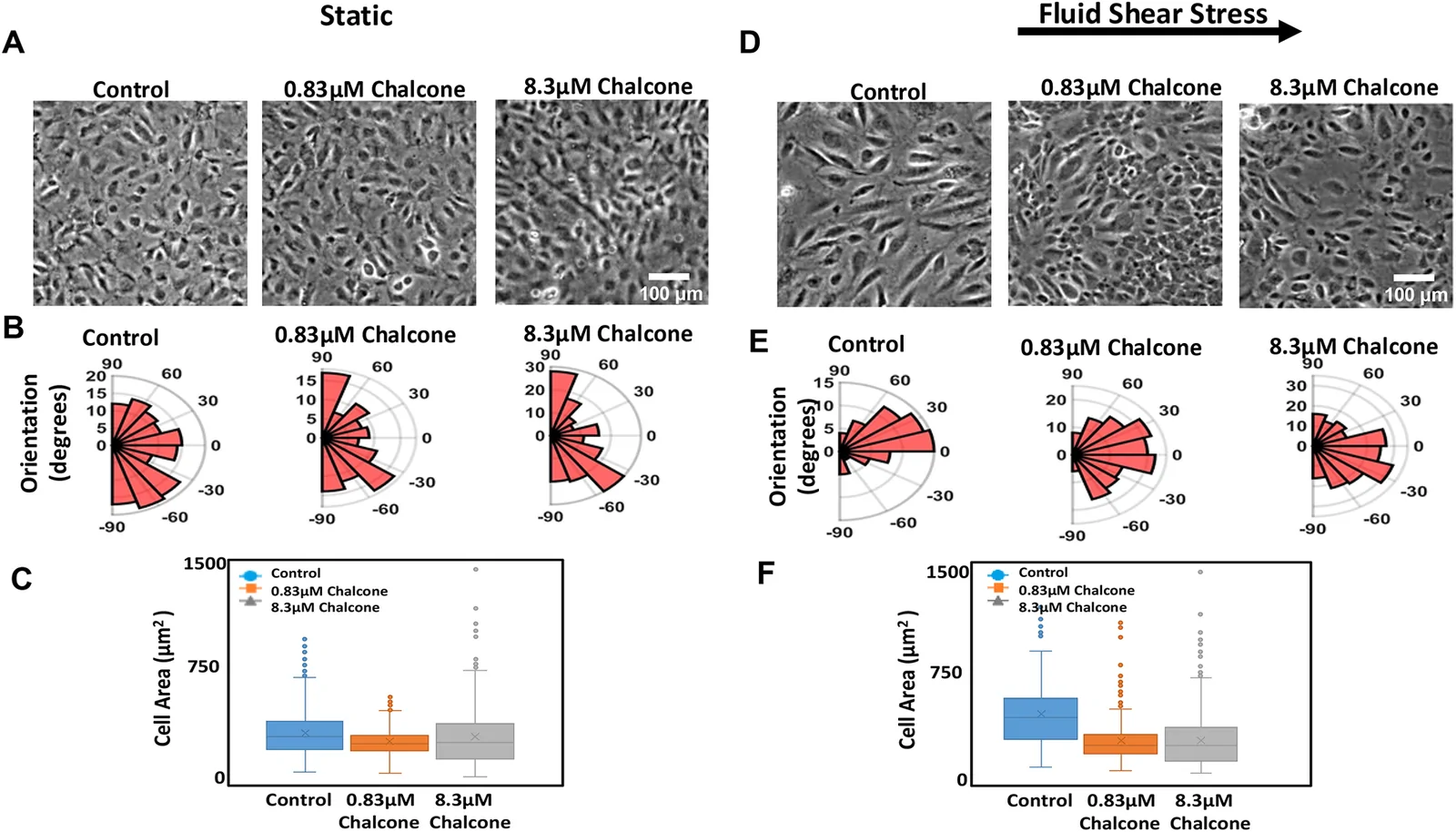
**Impact of chalcone on EC morphology.** (A-C) Phase contrast images (A), cellular orientation (B) and cell area (C) of HUVECs exposed to vehicle control and chalcone concentrations of 0.83 μM and 8.3 μM under static conditions. (D-F) Phase contrast images (D), cellular orientation (E) and cell area (F) of HUVECs exposed to vehicle control and chalcone concentrations of 0.83 μM and 8.3 μM under FSS conditions. Error bars are s.e.m. and all measurements were taken after 1 h of incubation. The box represents the 25–75th percentiles, and the median is indicated. The whiskers show the 1.5x IQR from the Q1 and Q3 values (Tukey plots). The mean is indicated by an ×. (*n*=100 cells).

To assess the impact of Cx43 modulation on EC morphology and migration, we analyzed cell area, orientation and velocity of HUVECs treated with chalcone and RA. Representative brightfield images used to analyze cells exposed to chalcone under static and FSS are shown in Fig. 3A,D. Chalcone treatment impaired endothelial alignment along the flow direction, as evidenced by a decrease in the percentage of cells oriented within ±45° of the flow axis (Fig. 3B,E). Low- and high-dose chalcone treatments also reduced cell area by 18% and 9%, respectively, under static conditions, and by 40% and 39% under FSS (Fig. 3C,F).

**Fig. 4.**
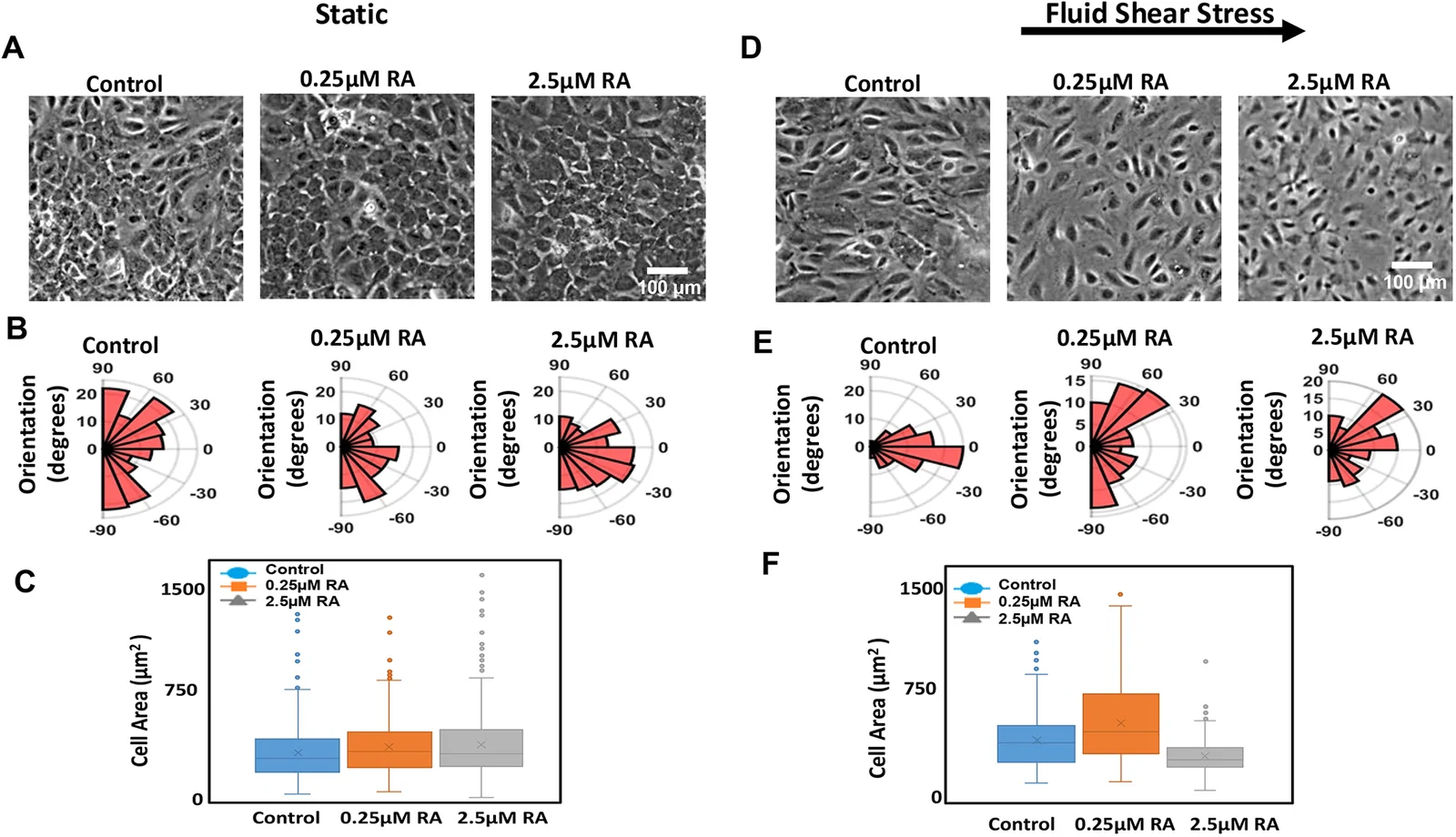
**Impact of RA on EC morphology.** (A-C) Phase contrast images (A), cellular orientation (B) and cell area (C) of HUVECs exposed to vehicle control and RA concentrations of 0.25 μM and 2.5 μM under static conditions. (D-F) Phase contrast images (D), cellular orientation (E) and cell area (F) of HUVECs exposed to vehicle control and RA concentrations of 0.25 μM and 2.5 μM under FSS conditions. Error bars are s.e.m. and all measurements were taken after 24 h of incubation. The box represents the 25–75th percentiles, and the median is indicated. The whiskers show the 1.5x IQR from the Q1 and Q3 values (Tukey plots). The mean is indicated by an ×. (*n*=100 cells).

In contrast, representative brightfield images used to analyze HUVECs exposed to RA under static and FSS are shown in Fig. 4A,D. RA treatment reduced the percentage of cells aligned along the flow direction compared to static control (Fig. 4B,E). Low-dose and high-dose RA increased cell area by 11% and 14%, respectively, under static conditions, whereas low-dose RA increased cell area by 29% and high-dose RA decreased it by 27% under FSS (Fig. 4C,F).

Analysis of cell migration velocities revealed that low-dose chalcone increased HUVEC velocity by 13% and 21% under static and FSS conditions, respectively, whereas high-dose chalcone decreased velocity by 50% and 10% under static and FSS conditions, respectively (Fig. S2A,B). RA treatment consistently reduced HUVEC velocity, with low-dose RA decreasing the velocity by 34% and 2% and high-dose RA decreasing the velocity by 44% and 38% under static and FSS conditions, respectively (Fig. S2C,D).

### Chalcone inhibition of EC GJIC is concentration dependent

**Fig. 5.**
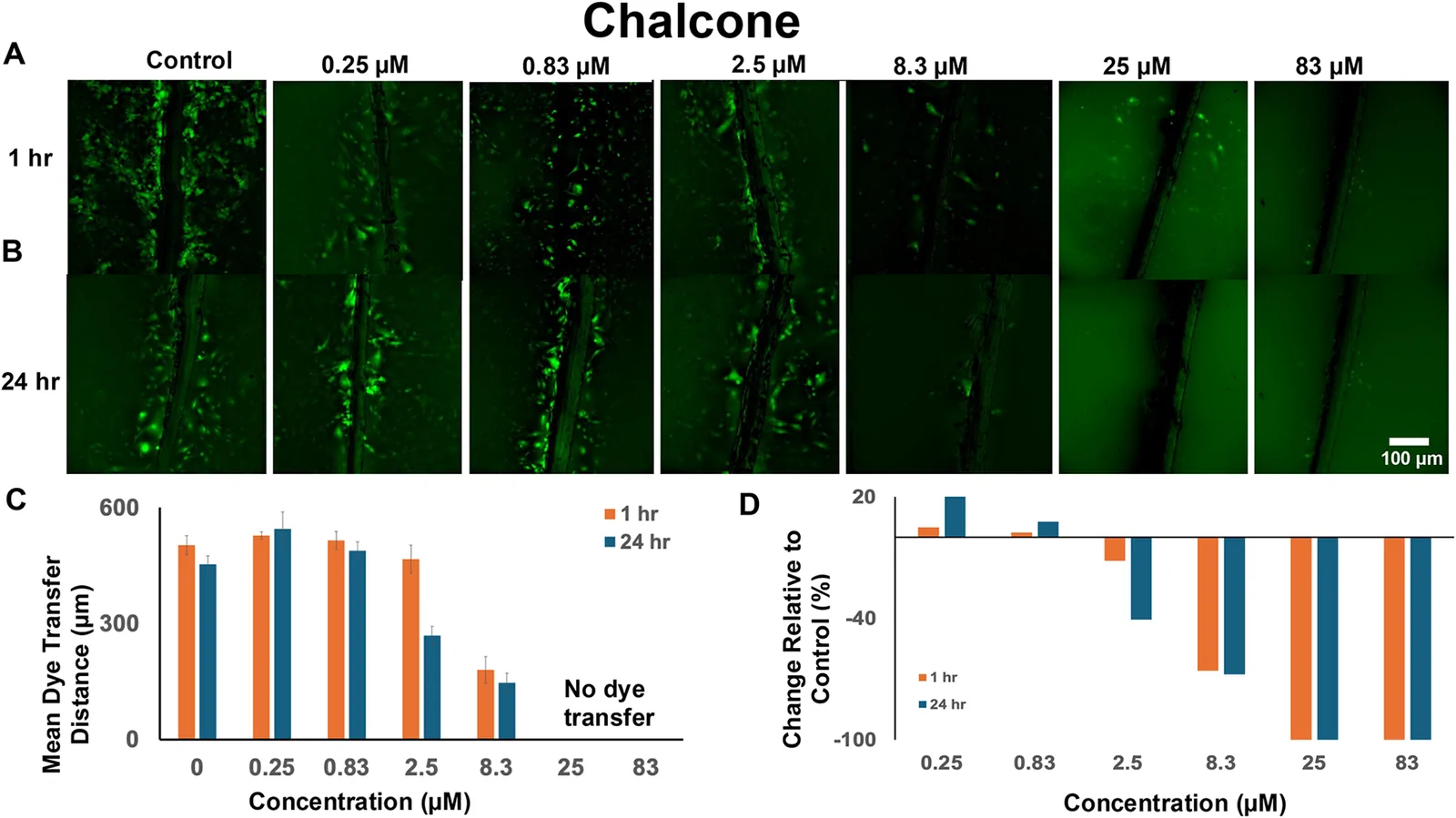
**GJIC after chalcone incubation.** (A,B) Fluorescent images of HAEC dye transfer after exposure to chalcone concentrations ranging from 0.25 μM to 83 μM at 1 h (A) and 24 h (B). (C,D) Mean dye transfer distance at 1 h and 24 h (C) and percentage change in mean dye transfer length relative to control (D). Error bars are s.e.m. (*n*=30 monolayers).

HAECs were exposed to increasing concentrations of chalcone ranging from 0.25 μM to 83 μM after 1 h and 24 h of incubation. After these incubation times, intercellular communication was assessed by GJIC. Relative to control, dye transfer was observed to remain relatively unchanged at lower chalcone concentrations (0.25 μM, 0.83 μM), but beginning at 2.5 μM dye transfer was observed to gradually decrease with increasing chalcone concentration (Fig. 5A,B). However, dye transfer was completely abolished beginning at 25 μM as no intact monolayers were observed beyond this time point. Quantification of the mean dye transfer length revealed an initial dye transfer length of 503±20 μm (control) that began to decline at 2.5 μM to 466±24 μm after 1 h of chalcone incubation (Fig. 5C). After 24 h of chalcone incubation, the decline in mean dye transfer length was more substantial decreasing from 453±24 μm (control) to 268±36 μm at 2.5 μM. Further quantification of the percentage change in mean dye transfer length relative to control revealed a small increase in mean dye transfer length at lower concentrations that began to decline with increasing concentrations beginning at 2.5 μM (Fig. 5D), which was observed to be independent of the times tested here (1 h and 24 h).

### The impact of RA on EC gap junction communication is concentration and time dependent

**Fig. 6.**
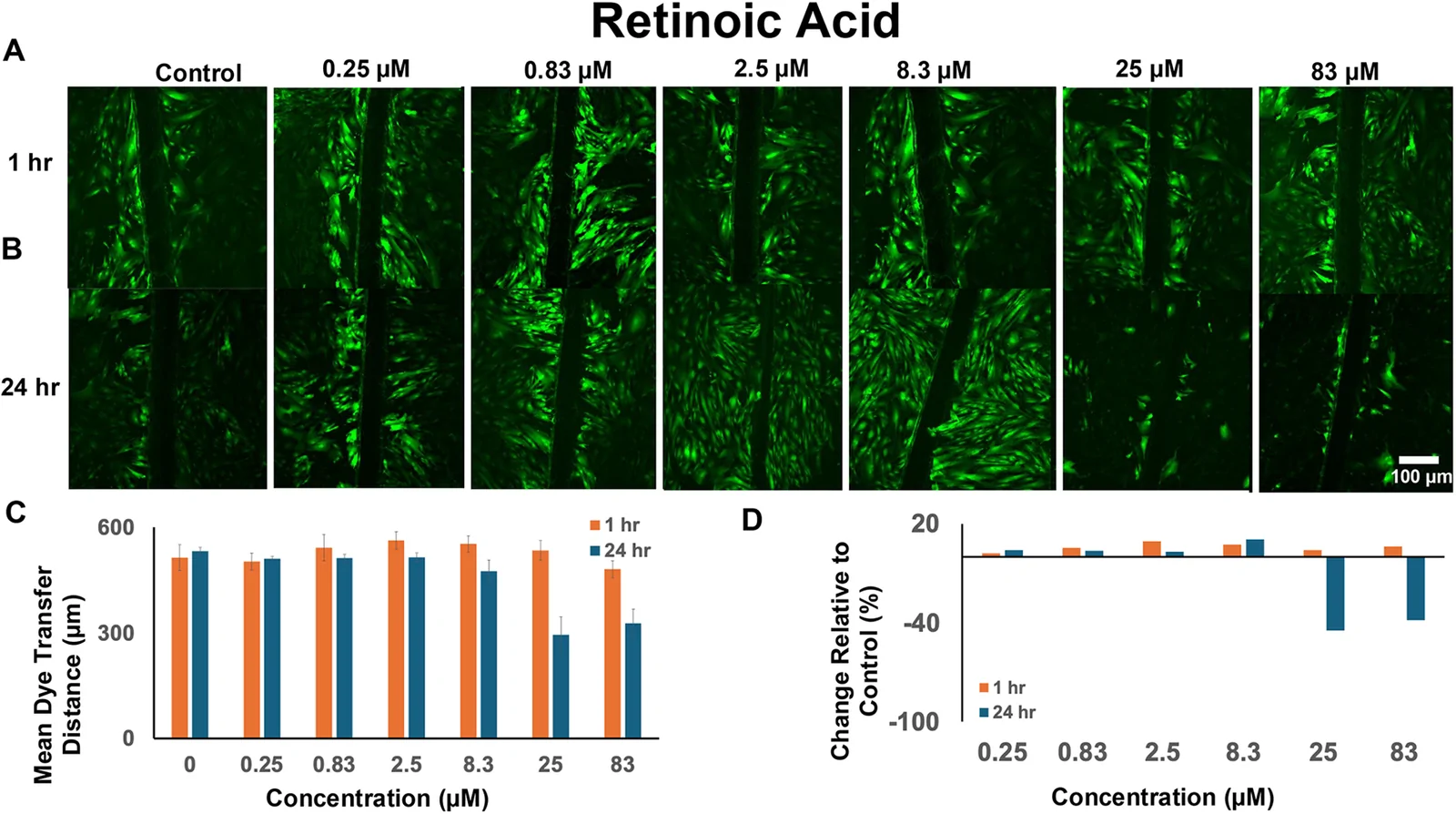
**GJIC after RA incubation.** (A,B) Fluorescent images of HAEC dye transfer after exposure to RA concentrations ranging from 0.25 μM to 83 μM at 1 h (A) and 24 h (B). (C,D) Mean dye transfer distance at 1 h and 24 h (C) and percentage change in mean dye transfer length relative to control (D). Error bars are s.e.m. (*n*=30 monolayers).

HAECs were exposed to RA concentrations ranging from 0.25 μM to 83 μM and GJIC was measured after 1 h and 24 h of incubation. Relative to control, dye transfer was observed to remain relatively unchanged throughout the entire range of concentrations mentioned above after 1 h incubation, but after 24 h dye transfer increased with increasing concentration up to 25 μM. At 25 μM and above, dye transfer was reduced (Fig. 6A,B). Quantification of the mean dye transfer length revealed a dye transfer length that fluctuated between 515±12 μm and 532±11 μm after 1 h of RA incubation (Fig. 6C). However, mean dye transfer length after 24 h of RA incubation revealed an initial dye transfer length of 513±37 μm, reaching values as high as 562.5±25 μm and as low as 295±51 μm, which was observed at 25 μM of RA (Fig. 6C). The percentage change in mean dye transfer length relative to control revealed a small increase in mean dye transfer length as RA concentrations increased, but a decline in mean dye transfer length was observed only at concentrations >25 μM and only after 24 h of incubation time (Fig. 6D).

### Chalcone reduces actin stress fibers and disrupts monolayer cohesion and Cx43 structure

**Fig. 7.**
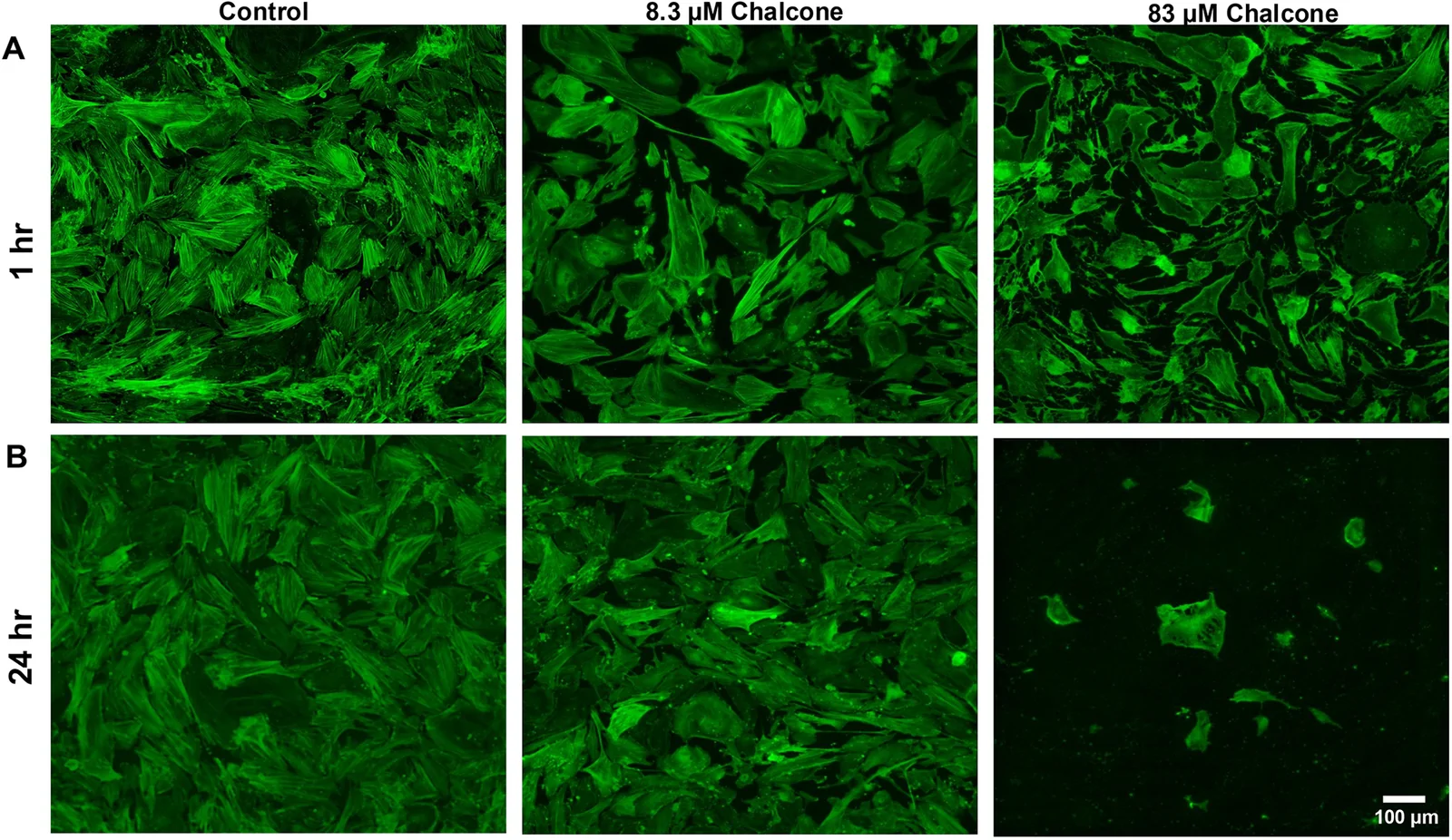
**Impact of chalcone on actin structure.** (A,B) Fluorescent images of F-actin after exposure to vehicle control and chalcone concentrations of 8.3 μM and 83 μM at 1 h (A) and 24 h (B). Images shown are representative of 50 repeats.

Upon incubation of HAECs to the range of chalcone concentrations mentioned above for GJIC (0.25-83 μM), F-actin staining revealed a reduction in actin stress fibers that began at 8.3 μM (Fig. 7) and continued to decrease with increasing chalcone concentration (Fig. S3A and S4A). This reduction in actin stress fibers was accompanied by an increase in visible gaps between cells, suggesting a reduction in monolayer cohesion. However, although the behavior reported above was time independent, we note that cells exposed to chalcone for 24 h exhibited the most dramatic and severe reductions in actin stress fibers and monolayer cohesion. In addition, Cx43 staining revealed an observable reduction in intensity as chalcone concentration increased as well (Figs S3B and S4B).

### Retinoic reduces actin stress fibers, monolayer cohesion, and Cx43 structure in a time- and concentration-dependent manner

**Fig. 8.**
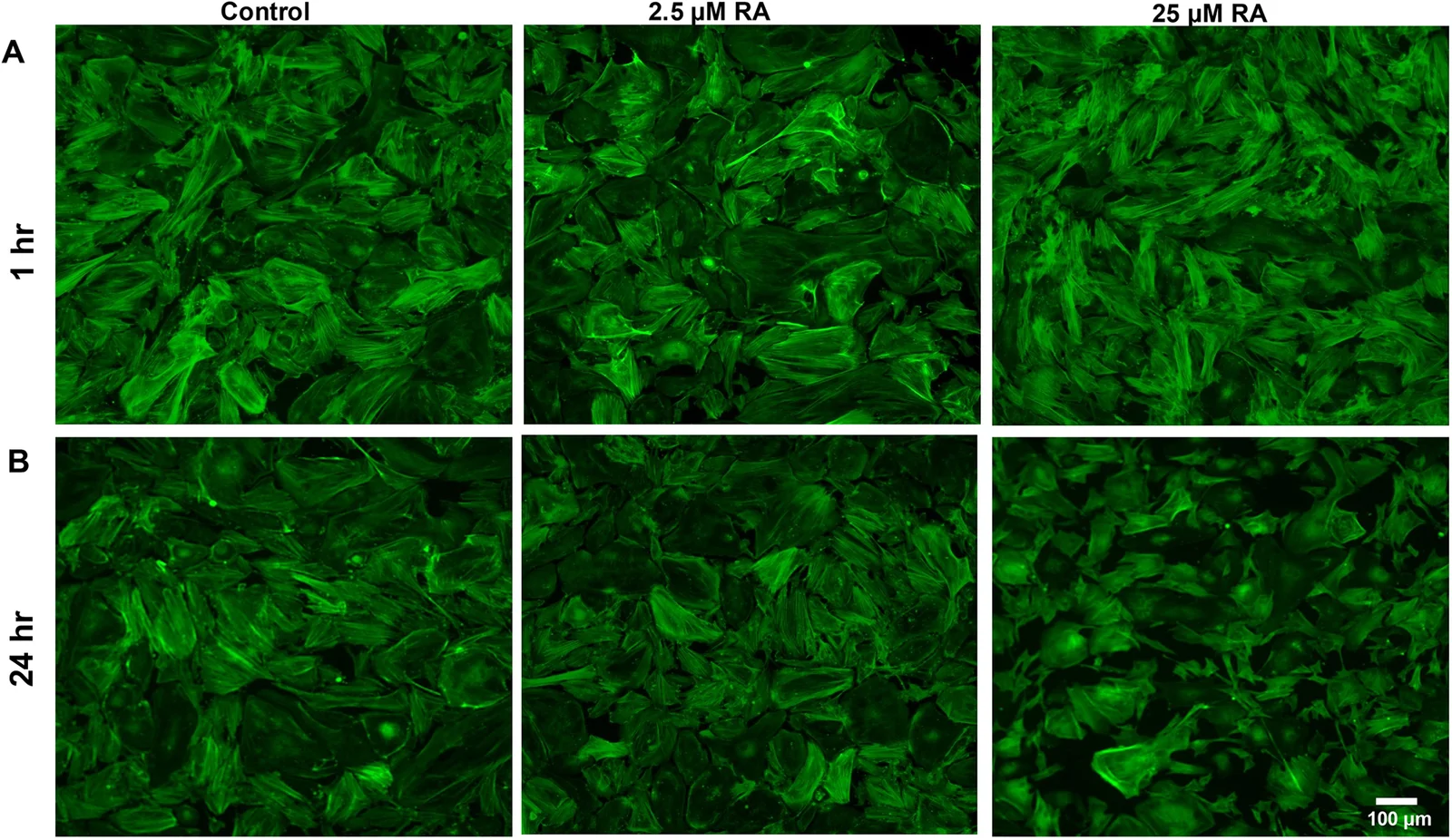
**Impact of RA on actin structure.** (A,B) Fluorescent images of F-actin after exposure to vehicle control and RA concentrations of 2.5 μM and 25 μM at 1 h (A) and 24 h (B). Images shown are representative of 50 repeats.

HAECs exposed to RA concentrations ranging from 0.25 μM to 83 μM displayed F-actin stress fibers that were structurally indistinguishable at all concentrations after 1 h of incubation (Fig. 8A, Fig. S5A). After 24 h of RA incubation, stress fiber density and monolayer cohesion increased beginning at 2.5 μM, but decreased beginning at 25 μM (Fig. 8B, Fig. S6A). The transient increase and subsequent decrease in actin stress fibers and monolayer cohesion reported above was accompanied by similar behavior of Cx43 structure (Figs S5B and S6B).

## DISCUSSION

Our data show that mild inhibition of Cx43 activity using a low dose of chalcone enhances tractions and intercellular stresses under both static and FSS conditions, while more pronounced Cx43 inhibition with a high dose of chalcone leads to a significant reduction in these biomechanical properties. Our findings suggest that the initial increase in cell-derived mechanical stresses upon moderate Cx43 inhibition may be a compensatory response mediated by other cell–cell junctions, such as tight junctions, which have been shown to colocalize with Cx43 in the vasculature (Nagasawa et al., 2006). However, further inhibition of Cx43 activity appears to compromise the ability of ECs to generate and maintain adequate tractions and intercellular stress, which may have implications on endothelial function. This notion is supported by previous studies that have suggested a link between vascular dysfunction in animal models of Cx43 deficiency (Tien et al., 2014). Furthermore, Depaola et al. and Ebong et al. reported laminar flow to (1) initially increase endothelial Cx43 expression, but return to control levels after prolonged exposure (DePaola et al., 1999) and (2) reduce Cx43-mediated GJIC (Ebong and DePaola, 2013). Our results support these two notions.

Interestingly, the effects of Cx43 upregulation on EC biomechanics were found to be flow dependent. Under static conditions, both low and high doses of RA reduced EC tractions, intercellular stresses, and strain energy compared to untreated controls. In contrast, under FSS, low-dose RA treatment enhanced EC biomechanical stresses, whereas high-dose RA consistently suppressed them. These results suggest that moderate Cx43 activity balanced with physiological FSS may act in a complementary manner to maintain endothelial function, whereas excessive Cx43 activation may have detrimental effects on the endothelium.

The observed changes in EC morphology and migration upon Cx43 modulation further highlight the complex interplay between gap junctions and EC mechanobiology. Chalcone treatment reduced EC area and impaired cell alignment along the flow direction, whereas RA treatment increased EC area and disrupted flow-induced cell alignment. These findings are consistent with previous studies reporting altered EC morphology and migration in response to Cx43 downregulation and upregulation (Kwak et al., 2001; Morbiducci et al., 2016; Tien et al., 2014).

Our GJIC and cytoskeletal analyses reveal that pharmacological modulation of Cx43 produces distinct concentration- and time-dependent effects on endothelial communication and structural cohesion. Chalcone-mediated Cx43 inhibition resulted in a progressive reduction in dye transfer beginning at intermediate concentrations, with complete loss of GJIC coinciding with monolayer disintegration at higher doses. This functional decline was accompanied by graded disruption of F-actin stress fibers, reduced Cx43 junctional staining, and increased intercellular gaps, indicating that Cx43 suppression may compromise both biochemical communication and mechanical integrity of the endothelial monolayer. Importantly, the onset of reduced GJIC at concentrations that preserved monolayer continuity suggests that loss of gap junction function precedes overt structural failure, supporting the notion that Cx43 plays an active role in maintaining endothelial cohesion rather than serving solely as a passive conduit for small molecules.

In contrast, RA-mediated Cx43 upregulation enhanced GJIC and actin organization in a temporally restricted and concentration-limited manner, revealing a biphasic response. One possible explanation for this biphasic response is that moderate RA exposure promotes optimal Cx43 expression, junctional assembly and cytoskeletal coupling, whereas excessive or prolonged RA signaling may trigger junctional remodeling, altered connexin turnover or cytoskeletal reorganization that ultimately destabilizes gap junction communication and monolayer integrity. Prolonged RA exposure promoted increased dye transfer, stress fiber density, and monolayer cohesion up to a threshold concentration, beyond which both communication and cytoskeletal organization deteriorated. This transient reinforcement of junctional and cytoskeletal architecture is consistent with a model in which moderate elevation of Cx43 stabilizes cell–cell coupling and mechanical continuity, whereas excessive Cx43 expression or prolonged signaling may disrupt junctional homeostasis. In addition, other groups have reported RA to both increase and decrease Cx43 activity in a time- and concentration-dependent manner. While the direct mechanism by which RA influences Cx43 expression is beyond the scope of this study, previous studies have suggested that RA influences Cx43 primarily through activation of nuclear RA receptors (RAR/RXR), which can transcriptionally upregulate gap junction expression via RA response elements, while also modulating Cx43 junctional assembly and turnover through downstream signaling pathways that regulate connexin phosphorylation and cytoskeletal coupling (Long et al., 2010; Noguchi et al., 1999; Tanmahasamut and Sidell, 2005; Watanabe et al., 1999). This is supported by the fact that Cx43 mRNA and protein levels have been reported to increase with increasing RA concentrations, while phosphorylation may increase or remain unchanged, depending on the cell type and incubation time (Carystinos et al., 2001; Long et al., 2010; Noguchi et al., 1999; Tanmahasamut and Sidell, 2005).

In general, although the F-actin staining presented here demonstrates that Cx43 modulation is associated with reduced stress fiber density (chalcone) and altered cytoskeletal organization (RA), we acknowledge that the mechanistic link between Cx43-mediated intercellular communication and actomyosin-based force generation remains inferential. Specifically, we did not directly assess phosphorylated myosin light chain 2 as a marker of actomyosin contractility, or vinculin as a marker of focal adhesion formation and maturation. Future studies employing phosphorylated myosin light chain 2 immunostaining, vinculin localization analysis and pharmacological inhibition of ROCK or MLCK would be needed to establish whether Cx43-dependent changes in gap junction communication directly regulate the actomyosin contractility axis in ECs.

Together, these findings support a nonlinear relationship between Cx43 expression, gap junction functionality and cytoskeletal organization, and provide a potential mechanistic context for our biomechanical observations by linking dose-dependent changes in endothelial tractions and intercellular stresses to underlying alterations in intercellular communication and structural integrity.

In summary, our findings demonstrate that Cx43 serves as a key regulator of endothelial biomechanical adaptation by coupling intercellular communication, cytoskeletal organization, and force transmission in a manner that is both dose dependent and flow sensitive. Pharmacological suppression or enhancement of Cx43 revealed nonlinear relationships between gap junction functionality and endothelial force generation, highlighting the importance of balanced connexin activity for maintaining monolayer cohesion and mechanical homeostasis. By integrating traction force microscopy, monolayer stress microscopy and functional assessments of gap junction communication, this study provides mechanistic insight into how perturbations in Cx43 expression translate into altered endothelial mechanics under static and shear stress conditions.

## MATERIALS AND METHODS

### Cell culture

HUVECs were purchased commercially from Fisher Scientific and cultured in Medium 200 (Thermo Fisher Scientific) supplemented with Large Vessel Endothelial Supplement (Thermo Fisher Scientific) and 1% penicillin-streptomycin (Corning). HAECs were purchased commercially from ScienCell and cultured in Endothelial Cell Medium supplemented with EC growth supplement, fetal bovine serum and 1% penicillin-streptomycin. All cells were cultured in 0.1% gelatin (Sigma-Aldrich)-coated flasks at 37°C and 5% CO_2_.

### Polyacrylamide gel fabrication

Polyacrylamide gels with a stiffness of 1.2 kPa were prepared as previously described (Islam and Steward, 2019a,b). Briefly, 35 mm Petri dishes (20 mm well; Cellvis) were treated with a bind silane solution for 45 min, rinsed with DI water, and air-dried before hydrogel polymerization. Polyacrylamide gel solution was prepared by mixing ultrapure water, 40% acrylamide (Bio-Rad) and 2% bis-acrylamide (Bio-Rad). Prior to gel polymerization, 0.5 μm diameter red fluorescent carboxylate-modified microspheres (Invitrogen) were added to the gel solution. The solution was degassed for 40-45 min and, subsequently, 10% ammonium persulfate and N,N,N′,N′-tetramethylethane-1,2-diamine (TEMED) was added to polymerize the gel on the treated Petri dishes. The gels were then flattened using 18 mm circular coverslips (Thermo Fisher Scientific), yielding a gel height of 100 μm.

### Cellular micropatterning

Polydimethylsiloxane micropatterns were fabricated as described previously (Islam and Steward, 2019a,b). Polydimethylsiloxane micropattern stamps with 1.25 mm circles were placed on top of polyacrylamide gels, which were treated with Sulfo-SANPAH (Proteochem) and placed under a UV lamp for 8 min. After SANPAH treatment, micropatterned holes were rinsed with PBS and then coated with 0.1 mg/ml of collagen I (Advanced Biomatrix) overnight at 4°C. The following day, collagen was removed, and HUVECs were seeded at a concentration of 50×10^4^ cells/ml and allowed to attach for at least 1 h. After this time, micropatterns were carefully removed, and adherent cells were allowed to form confluent, circular monolayers for at least 24 h prior to experimentation.

### 2′,5′-Dihydroxychalcone and RA treatment

2′,5′-Dihydroxychalcone (chalcone) and RA (Sigma-Aldrich) were used to downregulate and upregulate Cx43, respectively. Stock solutions of chalcone and RA were dissolved in DMSO and subsequently diluted to the final concentrations used in this study: 0.25-83 μM. Final DMSO concentrations were maintained at 0.1% for all incubation concentrations. All vehicle controls were incubated with 0.1% DMSO for the specified incubation times. Endothelial monolayers were exposed to chalcone and RA for either 1 h or 24 h.

### FSS experiment

HUVEC monolayers were seeded in a sticky Slide I Luer flow chamber (Ibidi). This laminar flow chamber, along with a peristaltic flow pump, circulated cell culture media bubbled with 5% CO_2_ through the flow chamber, producing a laminar shear stress of 1 Pa.

### Time-lapse microscopy

Phase contrast and fluorescence images were taken every 5 min using a ZEISS inverted microscope (ZEISS Axio Observer Z1) with a 10× objective and a Hamamatsu camera. The RA-treated, chalcone-treated and untreated monolayers were imaged for 6 h at 5 min intervals.

### Traction force microscopy and monolayer stress microscopy

Traction force microscopy and monolayer stress microscopy were used as described previously by our group and other groups (Butler et al., 2002; De Pascalis and Etienne-Manneville, 2017; Islam and Steward, 2019a; Tambe et al., 2013; Trepat et al., 2009). Briefly, cell-induced substrate gel deformations were calculated using a particle image velocimetry routine custom-written in MATLAB, and cell-substrate tractions were calculated using Fourier transform traction force microscopy (Butler et al., 2002; Tambe et al., 2011; Trepat et al., 2009). Tractions results are presented here as root mean squared (RMS) tractions calculated as:

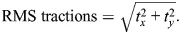
Intercellular stresses were then calculated using monolayer stress microscopy as described previously (Islam and Steward, 2019b; Tambe et al., 2011, 2013). In brief, monolayer stress microscopy uses a straightforward force balance leveraged from Newton's law to obtain a two-dimensional stress tensor within the specified cellular monolayer. By rotating the coordinate system, we computed the maximum principal stress (σ_max_) and minimum principal stress (σ_min_) with their respective orientations. At each point within the monolayer, we then computed average normal intercellular stress 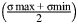 and maximum shear intercellular stress 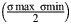. For analysis of tractions and intercellular stresses, a cropped 500×500 μm square was analyzed within the 1.25 mm circular monolayer.

### Immunohistochemistry

Cellular monolayers were fixed with 4% formaldehyde for 15 min at 37°C, followed by permeabilization with 0.2% Triton X-100 for 5 min. After permeabilization, cells were incubated with 2% bovine serum albumin blocking solution at 37°C for 45 min and subsequently incubated with a mouse monoclonal Cx43 antibody at a concentration of 1:400 (CX-1B1, Thermo Fisher Scientific) overnight at 4°C. The following day, HUVEC monolayers were incubated with Alexa Fluor 594 goat anti-mouse IgG (Thermo Fisher Scientific, A-11001) and Alexa Fluor 488 Phalloidin for 2 h. The cells were then mounted with Fluoromount-G with DAPI, sealed under a coverslip, and imaged after at least 24 h.

### GJIC

Intercellular communication was evaluated using the scrap loading dye transfer assay (Dydowiczova et al., 2020; Noguchi et al., 1999; Watanabe et al., 1999). In brief, after exposure to chalcone or RA at the concentrations and times mentioned above, cells were washed two or three times with PBS and subsequently scraped with a razor blade. Lucifer Yellow, diluted to a concentration 0.5 mg/ml, was added to the cells and incubated at 37°C for 5 min. After this time, excess Lucifer Yellow was removed from the cells, which were then rinsed an additional three or four times with PBS. After rinsing, the cells were fixed with 4% paraformaldehyde and imaged.

### Mean dye transfer length

Images of dye transfer were imported into ImageJ and mean dye transfer length was calculated by manually drawing horizontal lines perpendicular to the scrape area and measuring the maximum distance where dye transfer was observed. The mean dye transfer length represents the average length measured for each chalcone and RA concentration and exposure time.

### Cell area and cell orientation

Cell area and orientation were measured using a custom-written algorithm in MATLAB. This algorithm utilizes the image-processing toolbox and allows us to calculate the properties mentioned above by converting a phase contrast image to a binary image and subsequently segmenting this binarized image so that each individual cell can be identified. Each individual cell was assigned a number, and the cell area and cell orientation were extracted from our binary images using MATLAB. Our algorithm calculates the cell area as the number of pixels contained in a region (square pixels) and converts it to μm^2^ based on a pixel-to-micron conversion factor. The cell orientation was calculated by fitting an ellipse onto the cell body and calculating the angle between the major axis of the ellipse and the *x*-axis. Cell area and cell orientation code will be made available upon request.

### Statistical analysis

Statistical analysis was performed using single-factor analysis of variance (ANOVA) of different groups of ECs treated with RA and chalcone compared with the vehicle control. Statistical significance was set at *P*<0.05.

## Supplementary Material

10.1242/joces.264023_sup1 Supplementary information
