# Supplementary material for: Endothelial cell biomechanical adaptation to altered connexin 43 expression under fluid shear stress
Source: J Cell Sci. 2026 Aug 14;139(15):jcs264023. doi: 10.1242/jcs.264023 (PMC13535327; doi:10.1242/jcs.264023)
Supplement: Supplementary file 1 [file joces-139-264023-s1.pdf]

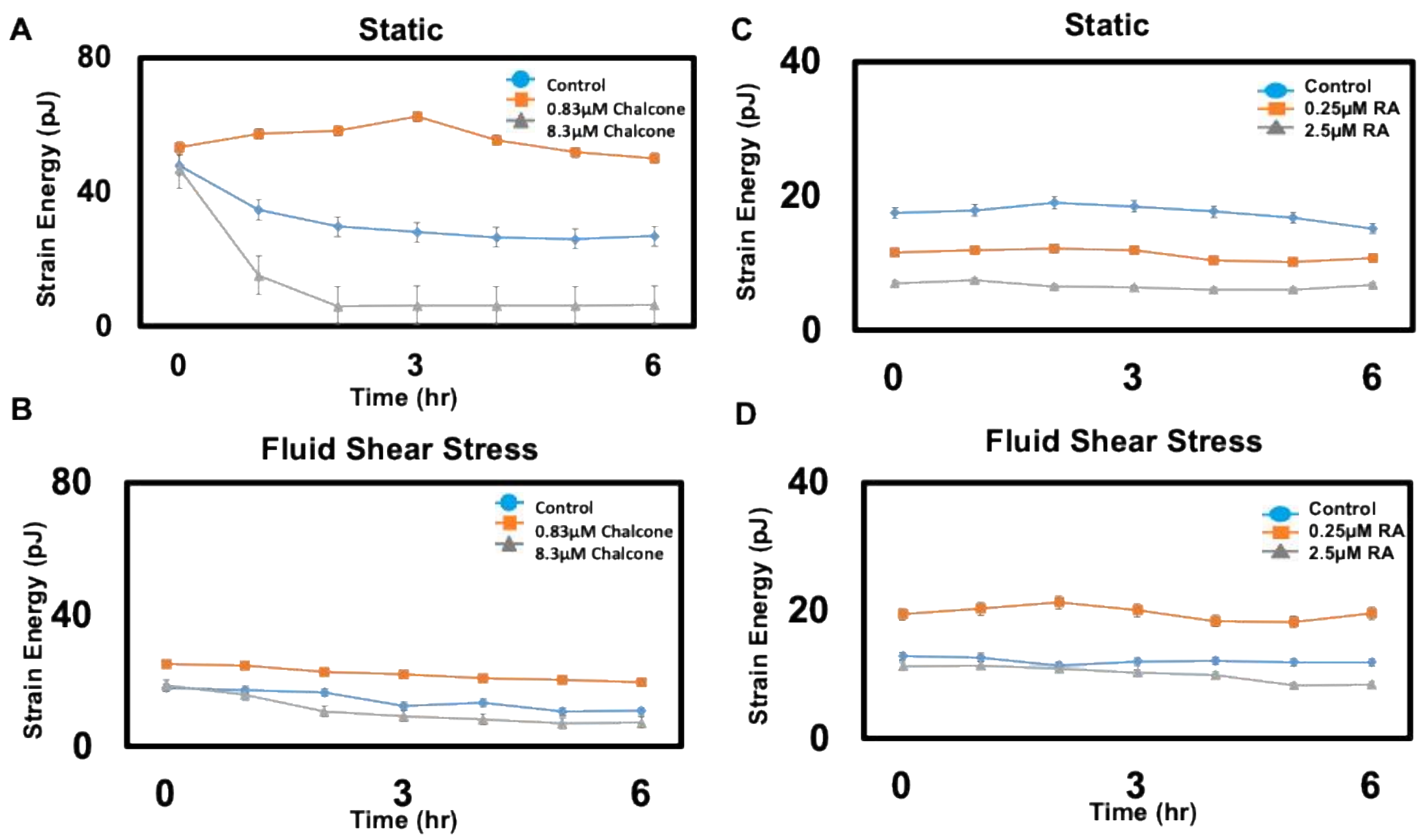

Fig. S1. Impact of chalcone and retinoic acid (RA) on endothelial stain energy. Strain energy of chalcone-treated HUVECs under static (PANOVA = 4.2E-12 < 0.05) (**A**) and fluid shear stress (PANOVA = 4.2E-12 < 0.05) (**B**) conditions. Strain energies of retinoic acid (RA)-treated HUVECs under static (PANOVA = 2.42E-11 < 0.05) (**C**) and fluid shear stress (PANOVA = 1.32E-10 < 0.05) (**D**) conditions. Error bars are standard error of the mean.

Journal of Cell Science • Supplementary information

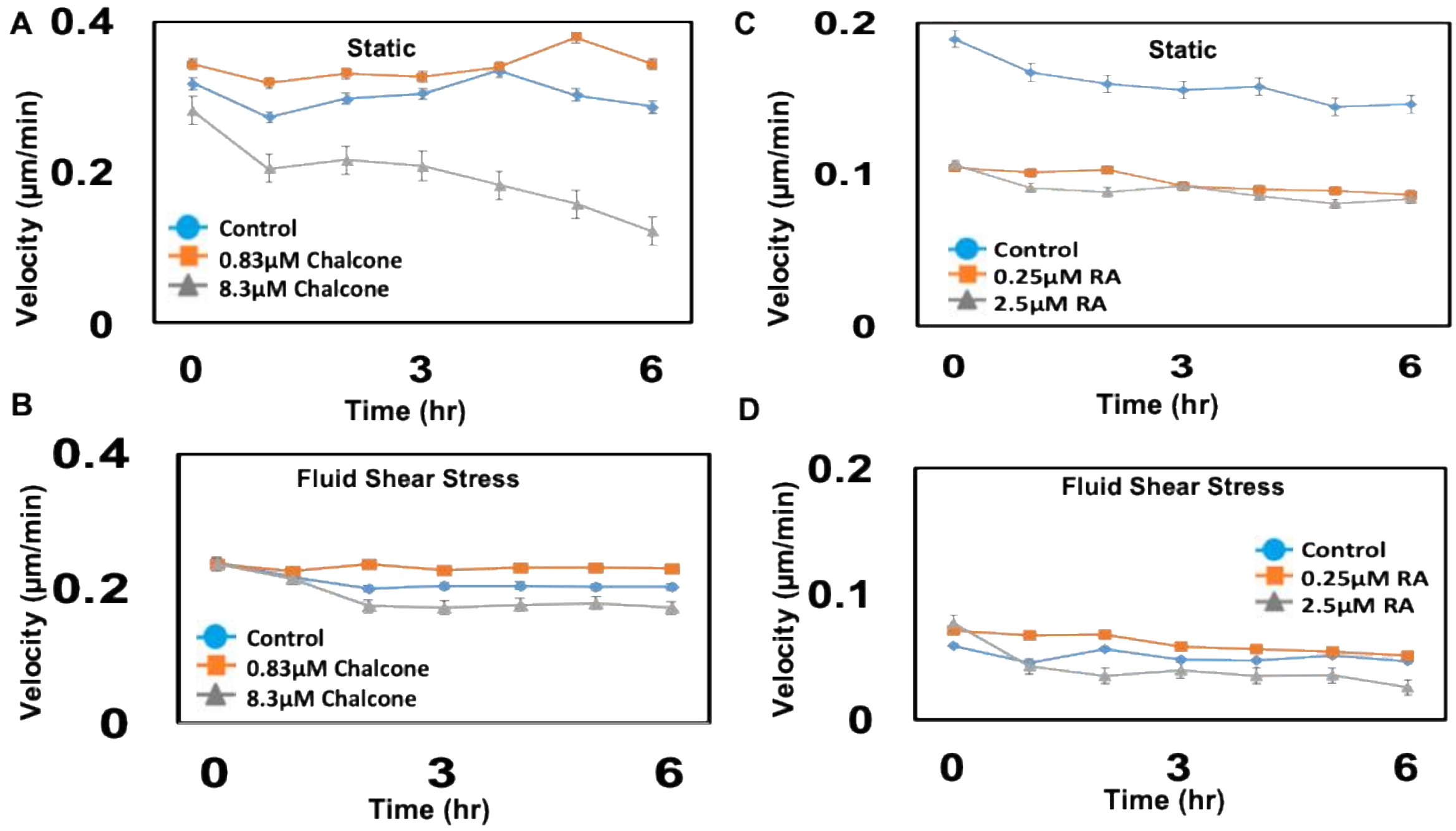

Fig. S2. Impact of chalcone and retinoic acid on cell velocity. Cell velocity of chalcone-treated HUVECs under static (**A**) and fluid shear stress (**B**) conditions. Cell velocity of RA-treated HUVECs under static (**C**) and fluid shear stress (**D**) conditions.

Journal of Cell Science • Supplementary information

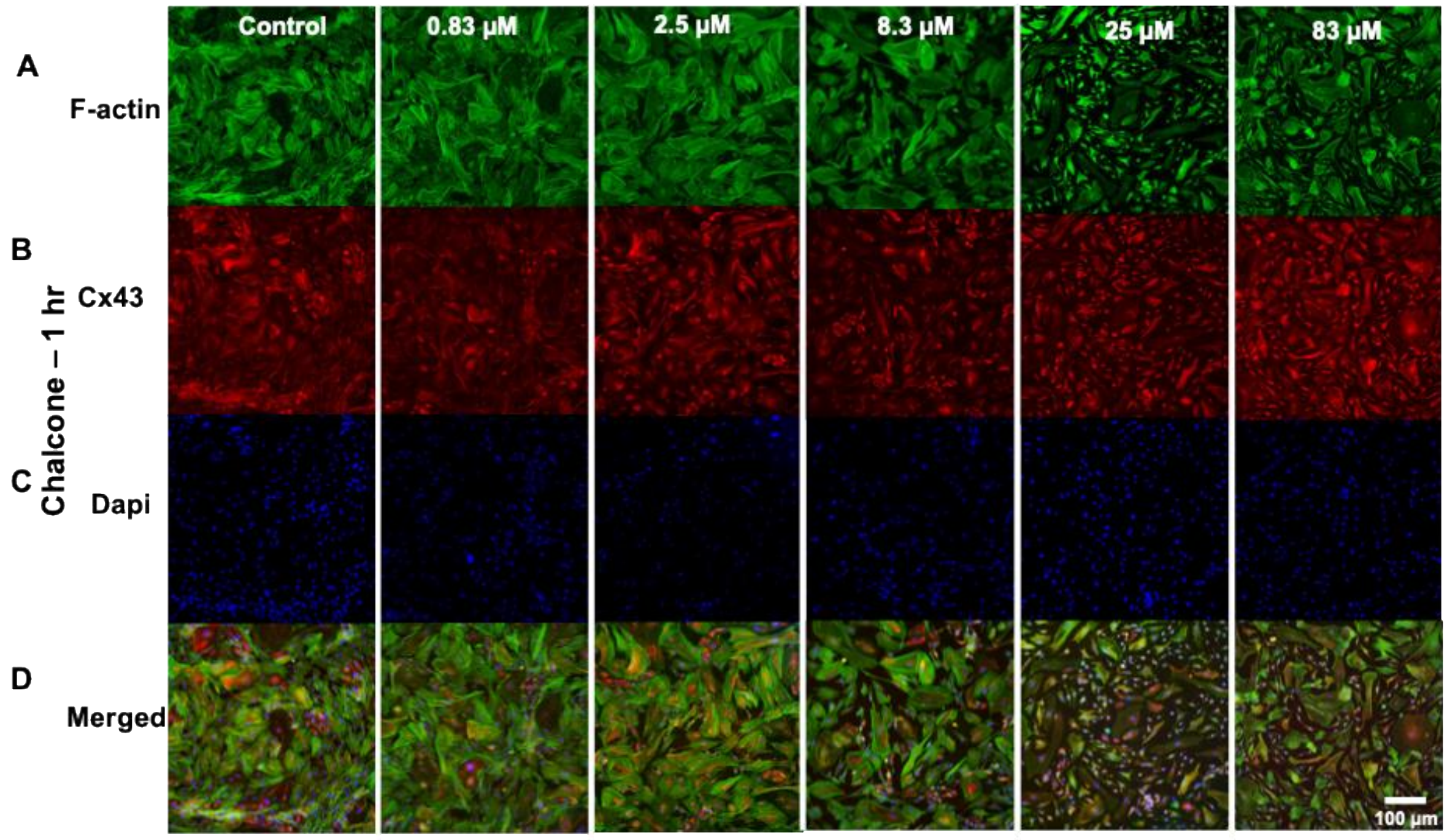

Fig. S3. Fluorescent images of f-actin (A), Cx43 (B), dapi (C) and merged (D) HAECs after exposure to chalcone after 1 hour.

Journal of Cell Science • Supplementary information

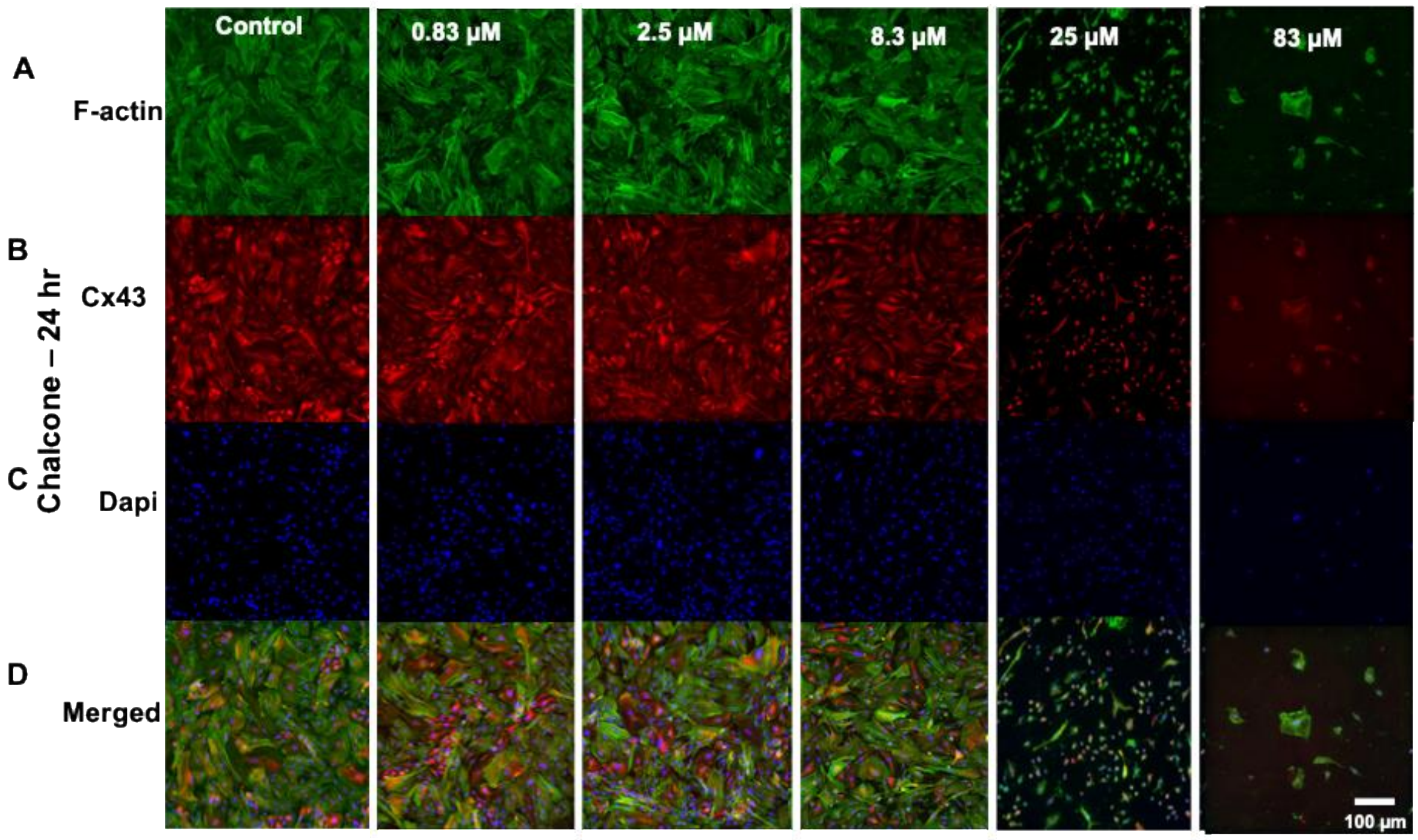

Fig. S4. Fluorescent images of f-actin (A), Cx43 (B), dapi (C) and merged (D) HAECs after exposure to chalcone after 24 hours.

Journal of Cell Science • Supplementary information

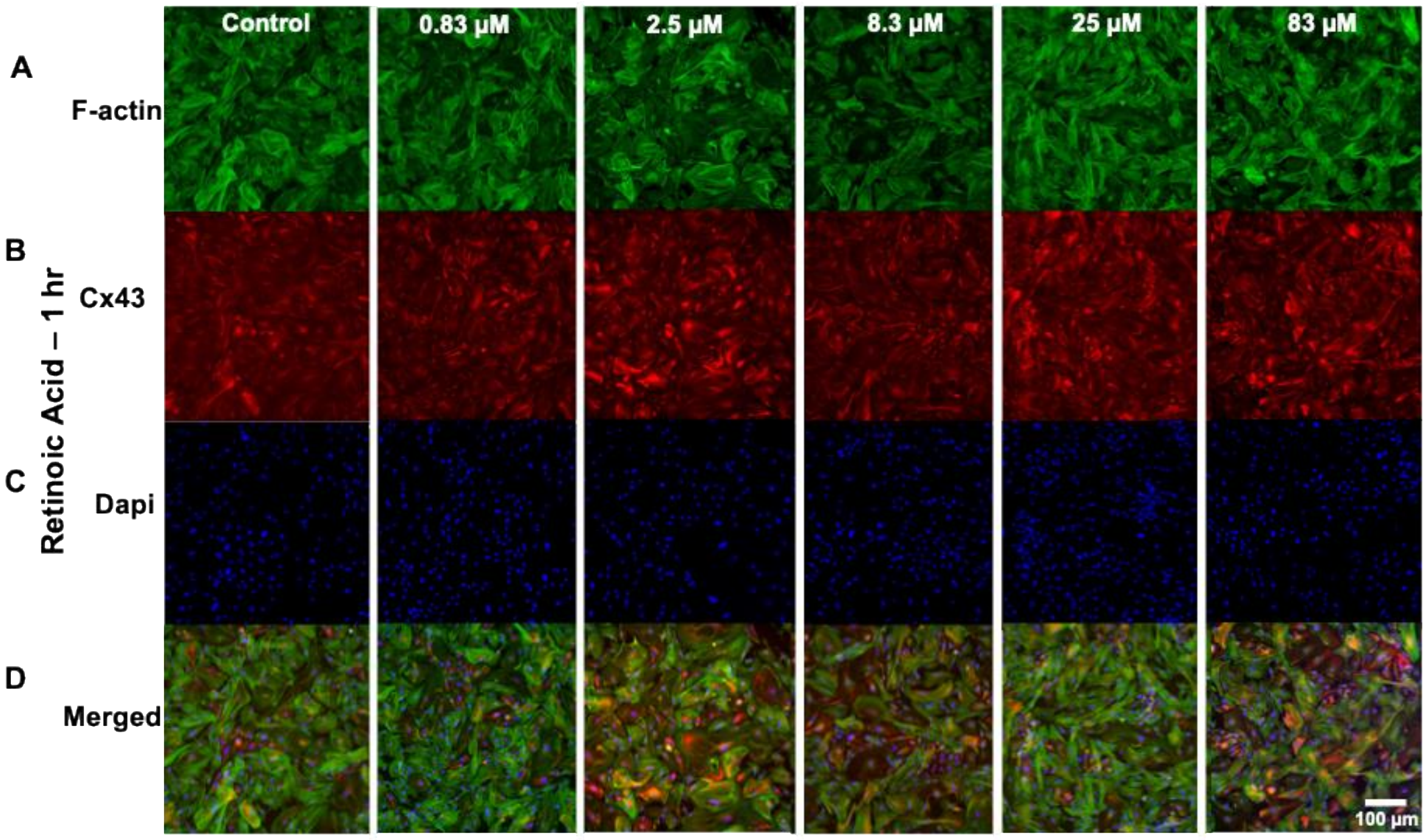

Fig. S5. Fluorescent images of f-actin (A), Cx43 (B), dapi (C) and merged (D) HAECs after exposure to retinoic acid after 1 hour.

Journal of Cell Science • Supplementary information

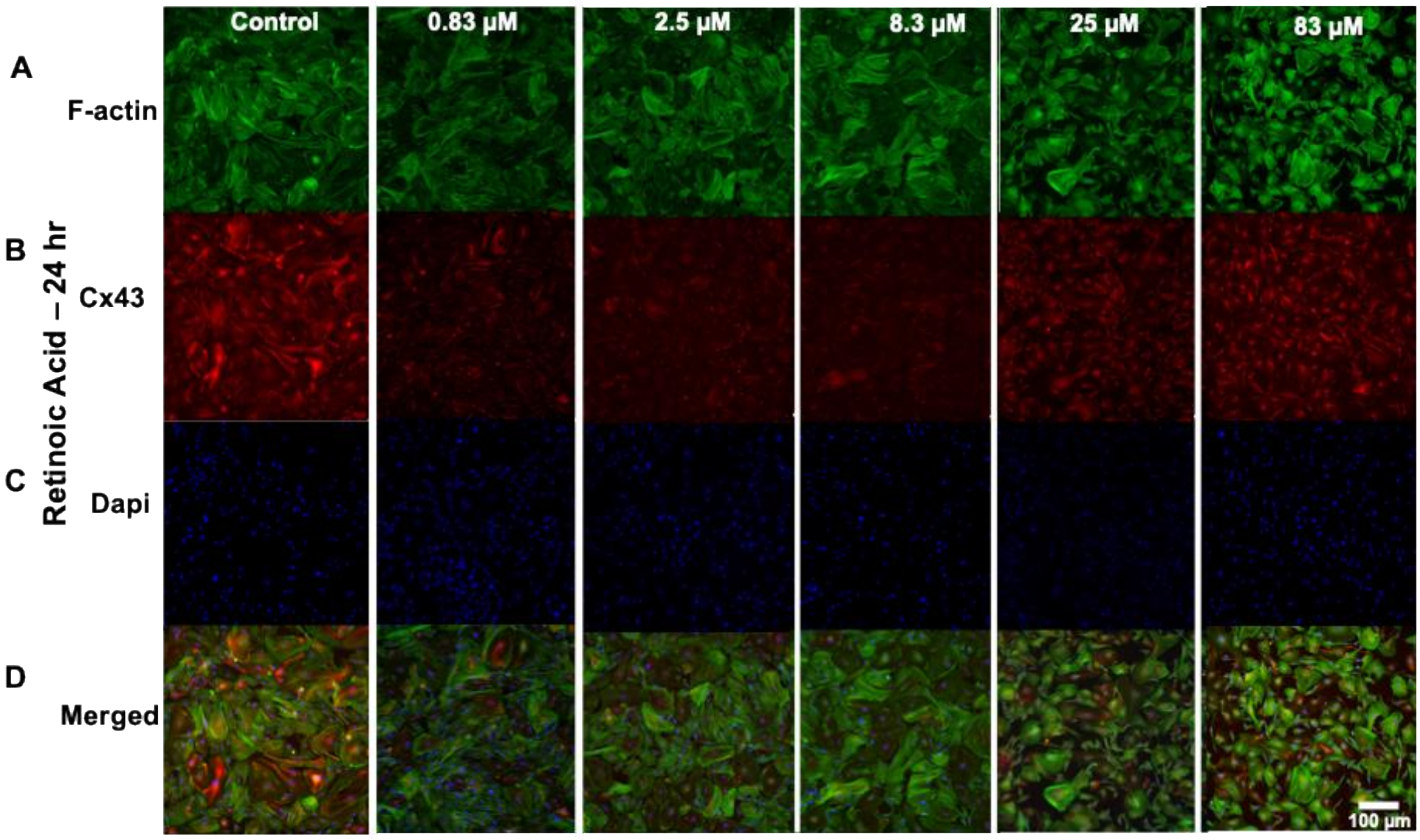

Fig. S6. Fluorescent images of f-actin (A), Cx43 (B), dapi (C) and merged (D) HAECs after exposure to retinoic acid after 24 hours.

Journal of Cell Science • Supplementary information
